# Supplementary material for: Nutritional Content According to the Presence of Front of Package Marketing Strategies: The Case of Ultra-Processed Snack Food Products Purchased in Costa Rica
Source: Nutrients. 2019 Nov 12;11(11):2738. doi: 10.3390/nu11112738 (PMC6893602; doi:10.3390/nu11112738)
Supplement: Supplementary file 1 [file nutrients-11-02738-s001.pdf]

Supplementary material

Definition and example of nutrition and health claims (30, 31)

Nutrition claim: Any representation which states, suggests or implies that a food has particular nutritional properties including but not limited to the energy value and to the content of protein, fat and carbohydrates, as well as the content of vitamins and minerals'

Types:

1. **Health ingredient claim**: "Any representation which states, suggests or implies that a food has particular nutritional properties not related to its energy value or to the content of protein, fat and carbohydrates, vitamins and minerals but related to the content of an ingredient".
   <u>Example:</u>

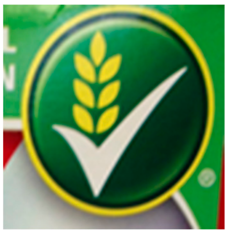

2. **Nutrient content claim:** "A nutrition claim that describes the level of a nutrient contained in a food or its energy value".
   <u>Example:</u> "Fortified with iron"

3. **Nutrient comparative claim:** "a nutrition claim that compares the nutrient levels and/or energy value of two or more foods".
   <u>Example:</u> "20% less fat".

"Health claims are any representation that states, suggests, or implies that a relationship exists between a food or a constituent of that food and health".

Types:

Nutrients **2019**, *11*, 2738; doi: 10.3390/nu11112738 www.mdpi.com/journal/nutrients

1. **General health claim:** "a health claim concerning the general beneficial effects of the consumption of foods or their constituents on health".

   Example:

   "Nutritious and delicious"

2. **Nutrient and other function claim:** Nutrient function claim is "a health claim that describes the physiological role of the nutrient in growth, development and functions of the body".

   Other function claims are "claims concerning specific beneficial effects of the consumption of foods or their constituents, in the context of the total diet on normal functions or biological activities of the body. Such claims relate to a positive contribution to health or to the improvement of a function or to modifying or preserving health."

   Example: "Vitamin D, essential for calcium absorption".

3. **Reduction of disease risk claim:** "health claims relating the consumption of a food or food constituent, in the context of the total diet, to the reduced risk of developing a disease or health-related condition."

   Example:
